# Lighting to Make You Feel Better: Improving the Mood of Elderly People with Affective Ambiences

**DOI:** 10.1371/journal.pone.0132732

**Published:** 2015-07-20

**Authors:** Andre Kuijsters, Judith Redi, Boris de Ruyter, Ingrid Heynderickx

**Affiliations:** 1 Human Technology Interaction Group, Industrial Engineering and Innovation Science, Eindhoven University of Technology, Eindhoven, the Netherlands; 2 Multimedia Computing Group, Intelligent Systems, Delft University of Technology, Delft, The Netherlands; 3 Philips Research, Eindhoven, The Netherlands; 4 Human Interaction with Intelligent Systems, Social sciences, Radbout University, Nijmegen, The Netherlands; University of Tuebingen Medical School, GERMANY

## Abstract

Current lighting technologies extend the options for changing the appearance of rooms and closed spaces, as such creating ambiences with an affective meaning. Using intelligence, these ambiences may instantly be adapted to the needs of the room’s occupant(s), possibly improving their well-being. We hypothesized that ambiences with a clearly recognizable, positive affective meaning could be used to effectively mitigate negative mood in elderly. After inducing a sad mood with a short movie one group of elderly was immersed in a positive high arousing (i.e., *activating*) ambience, and another group in a neutral ambience. Similarly, after inducing anxiety with a short movie one group of elderly was immersed in a pleasant low arousing (i.e., *cozy*) ambience, and another group in a neutral ambience. We monitored the evolution of the mood of the four groups of elderly over a period of ten minutes after the mood induction, with both self-reported mood measurements (every 2 minutes) and constant measurements of the skin conductance response (SCR) and electrocardiography (ECG). In line with our hypothesis we found that the activating ambience was physiologically more arousing than the neutral ambience. The cozy ambience was more effective in calming anxious elderly than the neutral ambience, as reflected by both the self-reported and physiological measurements.

## Introduction

Ambient intelligence research is devoted to designing systems that, embedded in our everyday environments, can help in improving the quality of our lives [1]. Among the various facets of ambient intelligence, a particularly interesting one is the so-called “empathic technology”: systems able to detect the affective state of a person, and react on it by providing the necessary care [2]. This type of systems is especially interesting for improving the life of vulnerable people, e.g., children, people with disabilities, or elderly. In our research, we focus on creating empathic technology for the elderly, going through a particularly difficult time of their life: the re-location to care centers.

Elderly in care centers often experience negative mood states [3,4]. They can be anxious because they realize they are in the last stage of their life, or sad because they miss their family, friends or pets [4]. Countering such negative moods is important, given that positive moods have been linked to increased health and well-being [5], and more prosocial behavior [6]. Human intervention (e.g., from care takers) is possibly the most effective way to improve elderly’s mood; however, often due to limited resources, human intervention is not always possible. As a consequence, equipping care centers with intelligent technology, able to detect negative moods and drive them towards more pleasant ones, may bring great added value to the well-being of care center inhabitants.

One way to achieve this is to use intelligent lighting solutions to create pleasant ambiences in care center rooms. The vast growth in the amount of LED sources, their miniaturization and the possibility to integrate sensors and electronics has created a wealth of options to automatically generate light of any color and intensity at any place or time with very thin, unobtrusive luminaries, making it possible to re-configure light according to a promptly detected negative mood of a room’s occupant. Light and color in general have been repeatedly shown to be able to influence people’s mood, both at a biological and psychological level. From a biological point of view, (short wavelength) light can influence the circadian rhythm, and as such the mood [7]. At a psychological level, (colored) lighting has been shown to carry meaning through associations, thereby influencing affective states. For example, employees working in colorful offices tend to experience more positive moods than employees sitting in neutral or colorless offices [8]. Different hues are also commonly associated with arousal levels: blue, for example, is deemed to be calming, whereas red is considered to be more energizing [9,10].

In addition, previous literature has shown that it is possible to use lighting to create ambiences whose affective connotation (also referred to as *atmosphere* [11], e.g. *cozy* or *activating*) can be clearly recognized by people [12,13]. This perceived affect may in turn impact the mood of the room's occupant. It is unknown, however, whether the exposure of a person to an affective ambience has the power to steer the person’s mood towards an affective state that matches the ambience connotation.

In this study, we hypothesize that affective ambiences, created with lighting, contribute to improve people's mood. We envision an Adaptive Ambience Creation platform that can automatically adapt the ambience in a care centre room to the needs of the elderly occupying it, and in this paper, we investigate its effectiveness. We regard mood as two-dimensional construct with valence and arousal as bipolar dimensions [14]. Valence (pleasure) summarizes how well one is doing, whereas arousal (activation) refers to a sense of mobilization or energy. We focus on countering, at a psychological rather than biological level, two negative moods commonly encountered in elderly just relocated in care centres, i.e. anxiety (negative valence and high arousal) and sadness (negative valence and low arousal) [3,4]. To do so, we resort to two affective ambiences, with an associated (and found to be recognized by elderly [12]) relaxing and activating atmosphere, respectively. These ambiences are created with the combination of general white lighting, varying in illuminance and color temperature, and colored accent lighting. We expect an ambience recognized as relaxing (more specifically, *cozy*) to lower arousal and improve the valence on an anxious elderly. Similarly, we expect an ambience recognized as *activating* to increase both arousal and valence of an elderly in a sad or gloomy mood.

This paper presents an experiment through which we provide evidence that carefully designed lighting ambiences with a clearly recognized positive affective connotation can help in improving the mood of elderly in the direction of the recognized affective connotation. To show this, we first induce a negative mood (i.e., either anxiety or sadness) in elderly participants. We then investigate whether a high arousing pleasant ambience (referred to as *activating*) can counter sadness more than a neutral ambience. Equivalently, we measure whether a low arousing pleasant ambience (referred to as *cozy*) can mitigate anxiety more than a neutral ambience. Specifically, we formulate these two hypotheses:

H1 An activating ambience is more effective in increasing both pleasure and arousal in elderly people that are in a sad mood than a neutral ambience.
H2 A cozy ambience is more effective in both increasing pleasure and reducing arousal in elderly people that are in an anxious mood than a neutral ambience.

**Fig 1:**
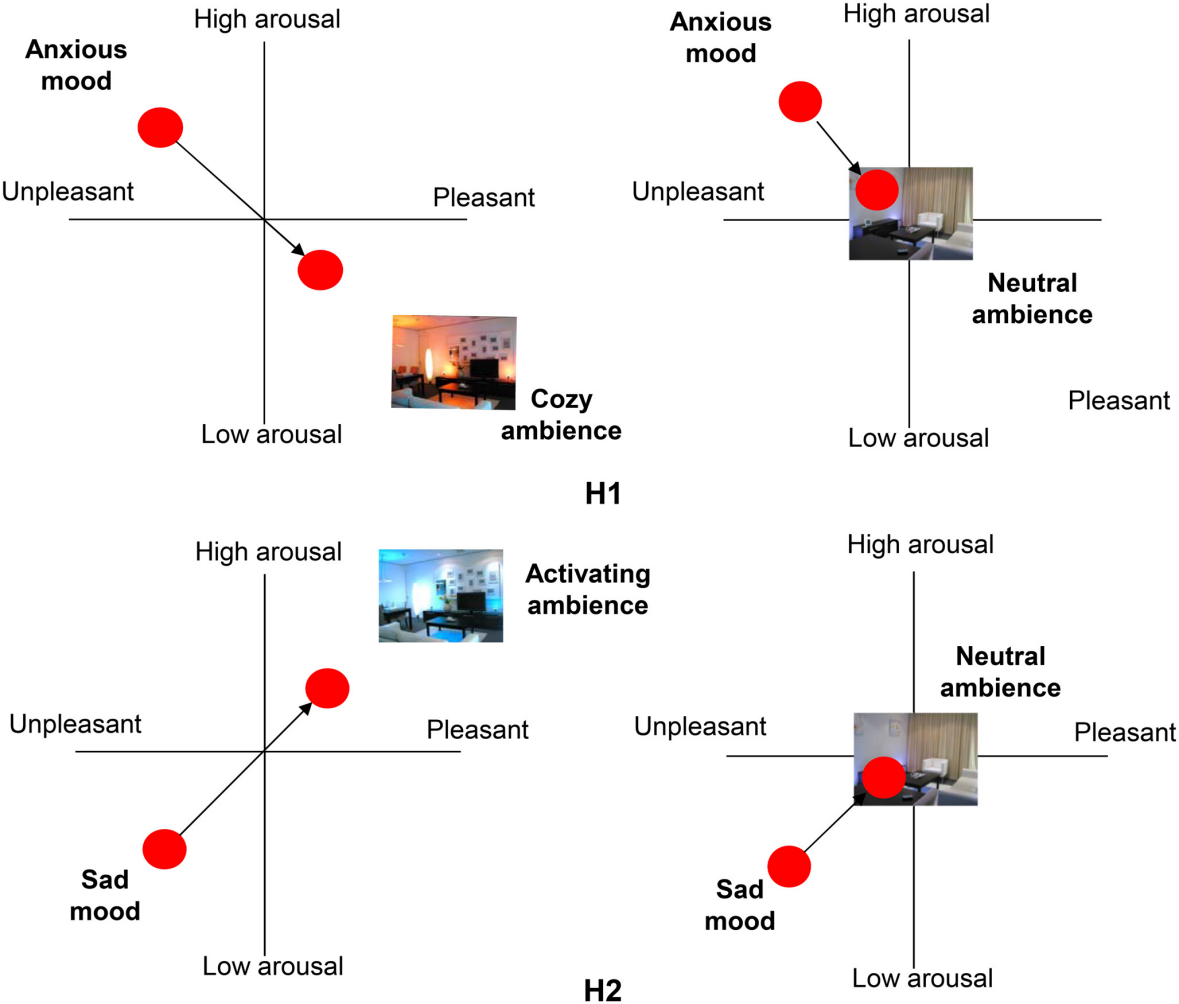
Schematic representation of the hypotheses formulated in this study. (H1) an activating ambience is more effective in increasing both pleasure and arousal in elderly that are in a sad mood than a neutral ambience, and (H2) a cozy ambience is more effective in both increasing pleasure and reducing arousal in elderly that are in an anxious mood than a neutral ambience.

Both hypotheses are visualized in Fig 1, and were tested by using self-reported and psychophysiological measures.

## Related Work

The relationship between lighting and affect has been studied from different perspectives and is largely documented; results are, however, not always coherent. We review results on the biological and psychological influence of lighting and color on mood. The biological (or non-visual) effects of light can be divided in indirect (via circadian photo entrainment) and direct effects on alertness and sleepiness [7,15]. The effects of light can be measured directly on melatonin or cortisol levels, but also via self-reported mood and physiological mood measures [15]. The psychological effects of lighting on mood are mediated by, amongst others, associations and preferences [10]. These effects are often measured with self-reported mood, but also with (psycho)physiological mood measures and via mood related behavior. In the last section we briefly discuss the existing knowledge on the affective connotation (i.e. atmosphere perception) of lighting ambiences.

### Influence of lighting on mood at a biological level

The indirect effects of lighting on mood are well documented [7,16,17]. Night time release of melatonin—a pineal hormone that promotes sleepiness and lowers the body temperature—can be phase advanced or delayed with the right exposure to bright light [18], thereby influencing the circadian rhythm of humans. Disruptions to circadian rhythms (e.g. due to jetlag, shift work, night time light exposure) can cause sleep and mood disorders [19–21]. Seasonal affective disorders (characterized by depression symptoms in the winter) are also considered to be the result of a disturbance of the circadian rhythm, caused by the failure to adapt to the shift in day length as a result of seasonal changes [19]. Bright light therapy of 1500 lx or more at eye level, has been shown to significantly reduce depression symptoms for people with seasonal mood disorders [21,22] or sleep disorders [21].

The wavelength of the light is also important; melatonin is especially suppressed by short wavelength light [23,24]. Thapan and colleques [24] revealed that melatonin was largely suppressed by blue light, slightly by green light and hardly or not at all by red light. This finding suggests that, at a biological level, blue light is more arousing than green light, and both are more arousing than red light.

Recent research also suggests direct effects of lighting on alertness and sleepiness [7,15,25]. Using different light spectra, studies reported the superiority of short wavelength light over long wavelength light in increasing alertness [7,15].

### Influence of lighting on mood at a psychological level

Along with biological effects, (colored) lighting has also strong psychological effects on the mood of people. Yoto and colleagues [26] found participants rating red colored cards as more arousing than blue colored cards. Kaiser [10] postulated that the arousing property of red may be mediated by associations with past experiences; red is often associated with danger and is often used as warning sign, while blue is associated with the sea or clear sky. In line with this argumentation, Küller and colleagues [9] revealed that a room painted red decreased EEG alpha activity of the participants more than a blue room, indicating an increased arousal in the red room. Earlier studies on the effect of colored lighting on the mood of people revealed similar results. Ali [27] revealed that red light suppressed EEG alpha activity more than blue light, indicating that red light was more arousing than blue light. Wilson [28] found that red light increased skin conductance compared to green light. Jacobs and Hustmeyer [29] found that red light projected on a screen was more arousing than green light, and green light more arousing than yellow and blue light, as measured by an increased skin conductance. Gerard [30] found that red light was more arousing than blue light, a conclusion that was deduced from an increased augmented systolic blood pressure, skin conductance, respiration rate, and cortical activation. He reported that red light yielded a variety of unpleasant associations involving blood, fire, danger as well as aggression, while blue light yielded associations with friendliness, blue skies, blue water of a lake or an ocean. These associations with colors might explain Gerard’s findings as he gave his participants the instruction: “If the colors bring certain thoughts and associations to your mind, allow yourself to have these thoughts, whatever they are” (p. 51) [30]. In contrast to the above findings, blue colored cards were found to be more arousing than red colored cards, as evidenced from subjective evaluations [31] and a lower electroencephalography (EEG) alpha and theta activity [26]. Nourse and Welch [32] found that test participants’ skin conductance response indicated more arousal when exposed to purple light compared to green light.

With respect to valence, it is often reported that short wavelength colors (such as blue, green) evoke more pleasant feelings than long wavelength colors (such as orange, red) [31,33,34]. This effect was found for office workers [34] and in shopping environments [33]. In contrast to these findings, Küller and colleagues [9] found that participants felt significantly more pleasant in a red room than in a blue room; this, however, was observed only in one of two reported experiments. Stone [35] found that the context was very important; positive mood increased in a blue open-plan study area compared to a red open-plan study area; however, opposite results were found for a private setting. In another study, Küller and colleagues [8] found that the presence of any color could positively influence people’s mood in work environments, although the affective response to colored walls was also depending on the context.

Not only the context but also the lightness and saturation of the color(ed lighting) was found to be very important for its influence on mood. Kaiser [10] argued that the green and red stimuli in the experiment of Wilson [28] were not equal in lightness and saturation level, and that the saturation differences might account for the findings. Also in the experiment of Nourse and Welch [32] the purple and green stimuli were not equal in saturation; the purple stimulus was more saturated. The higher saturation might be a better explanation for the higher skin conductance found in their experiment than the difference in hue. In line with this argumentation, Valdez and Mehrabian [31] revealed that the affective evaluation of colors was mainly based on saturation and lightness and less on hue. Similar results were found by Mikellides [36]; the saturation rather than the hue determined the arousing effect of a color. The lightness of a color was shown to increase arousal [15] and valence ratings [31]. Also saturation increased the valence ratings [31]; however, when the colors were too saturated, the effect became undesired. Kwallek and colleagues [34] found that highly saturated colors in an office induced more depression, confusion and anger with males.

The psychological effects of white light on mood have been studied extensively as well, revealing though mixed results. Some studies found no significant effect of white light on mood [37,38]. Although Baron and colleagues [37] did not find a direct effect of lighting settings on mood, they found several consistent effects of lighting on indirect measurements of mood. Warmer light (3000K) was found to enhance the willingness to help in a next experiment and to resolve conflicts as compared to cooler white light (4200K). Lower illuminance (150lx compared to 1500lx) was found to enhance ratings for an imaginary employee and to increase the amount of time subjects were willing to spend voluntarily on a next experiment. Knez and colleagues [39] argued that the exposure time in these studies was not long enough to induce mood changes. They performed several experiments [39–41] with a longer exposure time (i.e. 90 till 120 minutes). In their studies, females’ negative mood decreased while working under warmer light conditions (3000K) and increased under cooler light conditions (4000K), while an opposite effect was found for males; no significant effect was found for positive mood [39]. In a later study Knez and Enmarker [40] found an opposite result; females’ negative mood increased more under warmer white light (3000K), while males’ negative mood increased more in cooler white light (4000K). Females preserved their positive mood best under cooler white light (4000K), while males under warmer white light (3000K).

Finally, Küller and colleagues [8] investigated the effect of (indoor and outdoor) lighting on the mood of people working indoors in real work environments. Effects were measured in different seasons and different countries with different latitudes. Although they found no effect of illumination on the mood measurements, they found a strong correlation between mood and the appraisal of the lighting. If the lighting was judged right, self-reported mood was high, while mood decreased when the lighting was judged to be too dim or too bright. Similar results are reported by Veitch and colleagues [42]; people that judged the lighting in their room of higher quality reported a better mood.

### Atmosphere perception

The results described above reveal the high potency of lighting to reduce negative mood. However, drawing clear conclusions in terms of which light characteristic to use for a more positive mood is difficult as mixed outcomes are found, due to, amongst others., the discrepancy between psychological and biological influences, the context, and the interactions between the different lighting characteristics. In addition, it should be considered that people’s mood is not only affected by lighting, but also by other environmental factors (e.g., temperature) and non-environmental factors (e.g., internal state and personal preferences). In designing ambiences that can effectively improve the mood of elderly, it is therefore useful to first evaluate the perception of the ambience, i.e., the atmosphere. We argue that atmosphere perception mediates the psychological influence of lighting on mood.

Atmosphere is not an affective state in itself, but an affective evaluation of the environment [11]. Past studies have shown that atmosphere can be characterized along four dimensions, namely coziness, liveliness, detachment and tenseness [11], of which liveliness and coziness are the most important ones [11,43]. Alternatively, atmosphere may be evaluated in terms of valence and arousal [44,45]. The relation between both characterizations of atmosphere is that coziness was found to be positively correlated with positive valence and negatively correlated with arousal, while liveliness was found to be positively correlated with both valence and arousal [12]. Extensive knowledge has been built up about the influence of lighting characteristics on atmosphere perception [12,13,43,46]. The combination of general white lighting with colored accent lighting is known to be able to generate cozy, activating and exciting atmospheres in a room for younger people [12,47]. Despite the fact that atmosphere perception is different between younger and elderly people, it was possible to retrieve ambiences with a clear affective connotation for elderly too [12]. Nevertheless, it is so far unclear whether clearly recognized atmospheres have the power to influence the mood of people immersed in them, conditioning their mood towards matching the affective value of the atmosphere. This study is set out to verify whether that is the case.

## Method

To verify whether positive atmospheres could counter negative moods, we identified two negative moods commonly experienced by elderly in care centers [48], namely anxiety (i.e., negative valence and high arousal) and sadness (i.e., negative valence and low arousal), and two atmospheres with an opposite affective connotation, namely cozy (i.e., positive valence and low arousal) and activating (i.e., positive valence and high arousal) (see also Fig 1). To test the effectiveness of the cozy atmosphere we first induced an anxious mood. Then we exposed one group of participants to the cozy atmosphere and the other to a neutral one. We then monitored the mood of the participants in both groups for a period of 10 minutes. Our hypothesis was that we would find more positive valence and lower arousal (measured both self-reported and physiologically) for participants exposed to the cozy atmosphere than to the neutral one. The effectiveness of the activating atmosphere was tested in a similar way; inducing first sadness and then exposing one group to the neutral atmosphere and the other group to the activating one. Next we describe the details of the experimental setup.

### Participants

Thirty-eight older people, with an equal number of males and females, participated in the experiment. Their age varied between 66 and 94 years (M = 78.8 and SD = 6.8). They were recruited from the independent living unit of the Vitalis WoonZorg Groep in Eindhoven, The Netherlands. At the end of the experiment they were given a token of appreciation for their participation. This study was approved by the institutional review board of the Eindhoven University of Technology, following the Code of Ethics of the Dutch Institute for Psychologists, and by the ethical committee (ICBE) of Philips Research, following the Declaration of Helsinki, in 2013. All participants gave written informed consent.

### Experimental room

The experimental room was located at the Philips ExperienceLab and had dimensions of 7.4 x 4.2 x 2.8 meters. The walls were white, the ceiling had an off-white (i.e., ivory white) color, and the floor consisted of dark grey carpet tiles. The windows were blinded, in order to prevent influences of natural light during the experiments, and were covered with low chromatic curtains. The room was furnished as a living room with a white sofa and a white chair around a black coffee table. An off-white carpet was placed underneath the coffee table. Against the wall facing the sofa stood a black television cabinet with a black 42” television on it. Finally, a black dinner table with four off-white chairs were placed against the wall on the left-hand side of the sofa.

**Fig 2:**
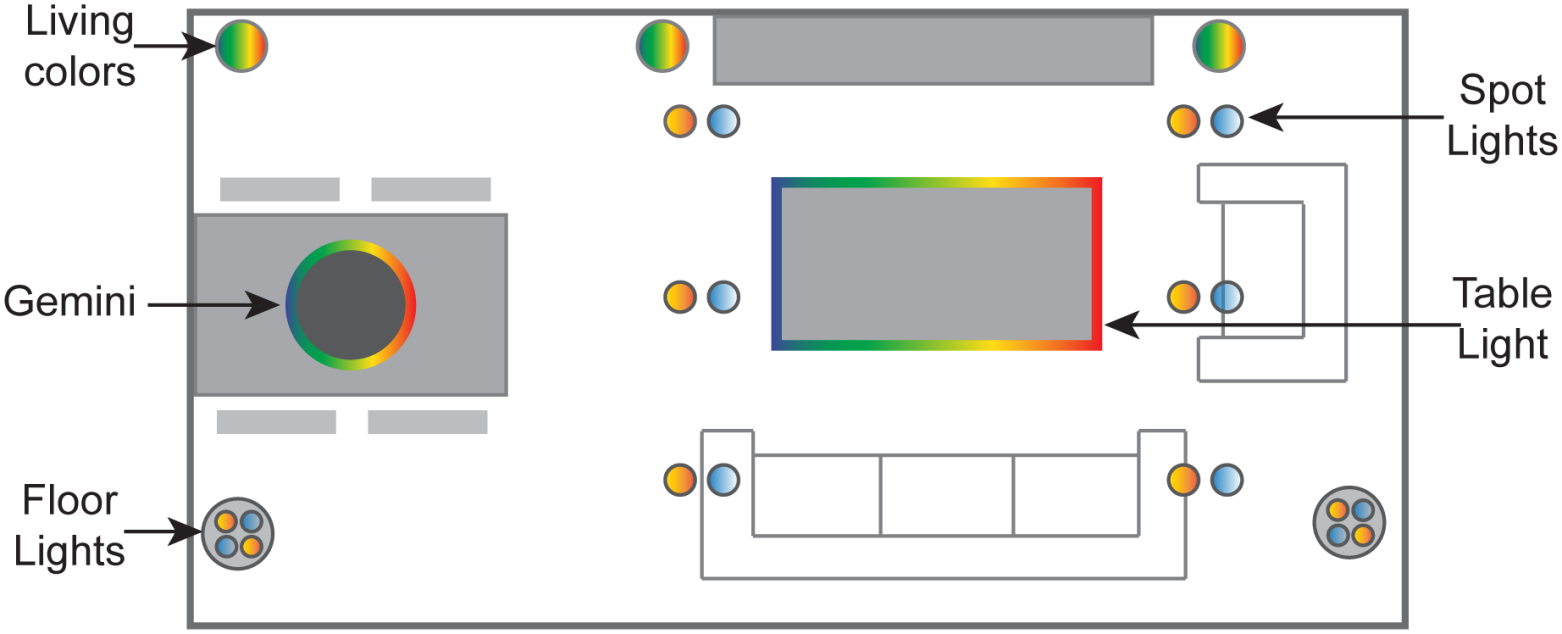
An overview of the installed luminaires in the experimental room.

An overview of the installed luminaires is given in Fig 2. Functional white lighting was provided by two cylindrical floor lights consisting of four fluorescent lamps each; two lamps with a warm white color temperature (CT) of 2700K (Philips Master TL5 HE 28W/827) and two lamps with a cold white of 6500K (Philips Master TL5 HE 28W/865 lamps). Additional white light was provided by six pairs of halogen spot lights; each pair consisted of one spot with a warm white CT of 3000K (Philips HR Dichroic 50W GU5.3 12V 36D) and one spot with a cool white CT of 4700K (Philips Diamondline 50W GU5.3 12V 36D 1CT). Decorative lighting was generated by three Philips Living Color lamps. Two were placed on each side of the television cabinet and one in the upper left corner. A table light consisting of red, green and blue LED strips was mounted underneath the coffee table and illuminated the floor locally. Finally, a Gemini lamp, consisting, on the one hand, of red, green and blue LEDs for illuminating the ceiling, and, on the other hand, of white LEDs for illumination downwards, was mounted above the dinner table.

### Ambiences

**Fig 3:**
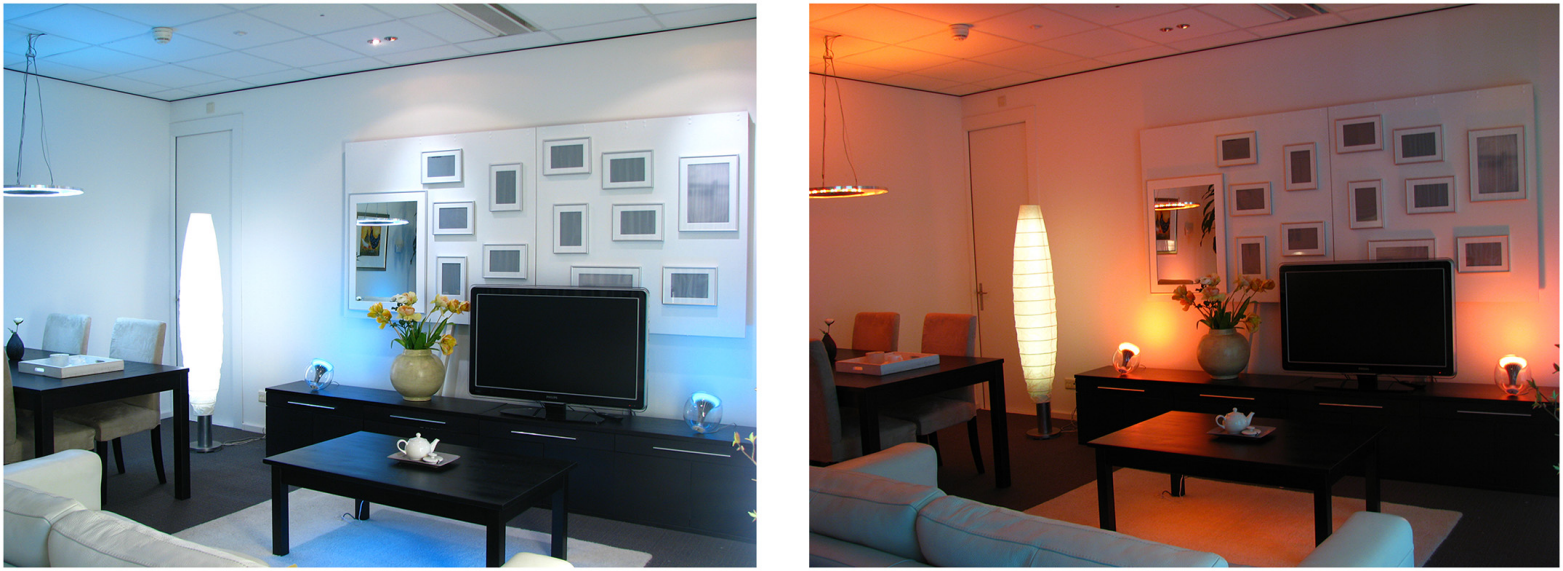
An impression of the activating ambience (left) and cozy ambience (right).

We used ambiences (pictured in Fig 3) for which we had confirmation that elderly could clearly recognize their atmosphere. These ambiences were designed and validated in a previous study [12]. The *cozy* ambience consisted of functional lighting at a lower illuminance (i.e., 120 lx) and lower CT (i.e., 2700 K), to which we added orange colored accent lighting. The Gemini lamp did not illuminate the dinner table in the *cozy* ambience. The cozy ambience was perceived as a pleasant low arousing ambience, as reflected by high coziness scores in the atmosphere questionnaire [12]. Coziness was found to be positively correlated with positive valence and negatively correlated with arousal [12]. The *activating* ambience consisted of functional lighting at a higher illuminance (i.e., 325 lx) and a higher CT (i.e., 4000 K), to which we added cyan colored accent lighting [12]. The activating ambience was perceived as a pleasant high arousing ambience, as reflected by high liveliness scores in the atmosphere questionnaire. Liveliness was found to be positively correlated with both valence and arousal [12]. The *neutral* ambience consisted of functional white lighting with an intermediate illuminance (i.e., 150 lx) and intermediate CT (i.e., 3400 K), and here we added white accent lighting. Pilot tests revealed that this ambience was scored neutral on both coziness and liveliness in the atmosphere questionnaire [11]. The reported illuminance and CT values were all measured vertically at the position of the participant’s eyes. More details about the design and validation of the cozy and activating ambiences can be found in [12].

### Mood induction

Different procedures for inducing mood in a controlled way exist (for reviews see [49–51]), and include listening to music, watching movies, watching affective slides, imagining scenes, and recalling past life events. For our study we chose a mood induction procedure based on viewing movie excerpts. This choice was based on the fact that induction with visual stimuli is less sensitive to demand characteristics [49], most effective in inducing the targeting negative mood states [49,51], and standardized material for the procedure is publicly available [52].

Two short film segments were selected from the database of Gross and Levenson [52]. For inducing sadness, the scene at which a boy cries at his father’s death (2’51”) from the movie ‘The Champ’ was selected [53]. For inducing anxiety, the basement chase scene (3’29”) from the movie ‘The silence of the lambs’ was selected [54].

### Mood measurements

The participants assessed their actual mood state with the pleasure and arousal scale of the Self-Assessment Manikin (SAM) [55]. Nine-point visual scales were used to assess the subjective level of pleasure and arousal of the participants.

During the trials we also monitored the electrodermal activity (via the skin conductance response, SCR) and the cardiac response (via the ECG signal) of the participants. Heart Rate (HR) and Heart Rate Variability (HRV) were computed from the ECG signal. Skin conductance response and HR increase are mainly linked to increased arousal [56–59]. Increased HRV is an indicator of an increased parasympathetic activity (i.e., resting state) and/or decreased sympathetic activity (i.e., active state). An increase in the high frequency component of HRV has been linked to an increase in parasympathetic activity.

The physiological responses were measured with the Nexus-10 system (Mind Media BV, Roermond, The Netherlands). The device was controlled with the Biotrace software suite (Mind Media BV, Roermond, The Netherlands) via a computer. The skin conductance response was recorded with the Nexus-10 SC/GSR sensors. The sensors were positioned to the upper phalanges of the index and middle finger of the non-dominant hand. For the ECG recordings, pre-gelled silver chloride electrodes were placed at the inside of the wrists of both arms, and a third electrode, functioning as ground sensor, was placed at the upper arm. The saving rate and resolution of the physiological measurements were: 32 Hz and 0.001 μS, respectively for SCR, and 2048 Hz and 1 μV, respectively, for ECG. Heart Rate (HR) and Heart Rate Variability (HRV) were computed offline from the ECG recordings by analyzing the variability in the intervals between sinus rhythm heart beats (i.e., R–R intervals), following the guidelines of the task force of the European Society of Cardiology and the North American Society of Pacing and Electrophysiology.[60] Custom software was written in MATLAB for the R-wave detection and the complete signal was carefully visually inspected for false or undetected R-waves, ectopic beat errors, and movement artefacts, which were then manually corrected. HR (beats per minute) was calculated from the inter-beat-intervals. Time and frequency domain HRV measures were analyzed using KARDIA [61] in MATLAB. The RMSSD (i.e., the square root of the mean squared differences of successive R-R intervals) was calculated as indicator of short term HRV. The High Frequency component (HF-HRV; 0.15–0.40 Hz) of HRV was calculated in the frequency domain with Fast Fourier Transform analysis (resample rate = 2 Hz, FFT window length = 512). The HF-HRV is a specific indicator of parasympathetic activity and is expressed in absolute values of power (in ms^2^).

### Procedure

The participants were welcomed in the experimental room and asked to sit on the sofa three meters in front of the 42 inch television screen. Each participant conducted two sessions; one session with a sad mood induction and one session with an anxious mood induction. The order of the mood induction was randomized. After the mood induction the influence of the ambience was investigated between subjects (Activating versus Neutral ambience after sad mood induction; Cozy versus Neutral ambience after the anxious mood induction). The order of the ambiences was also randomized resulting in four groups of experimental conditions (as also explained in Table 1). We counterbalanced male and female participants in each group.

**Table 1:** Distribution of participants over the experimental conditions; in the ‘Session1’ and ‘Session 2’ columns the first adjective refers to the induced mood, and the second adjective to the atmosphere used; for example, "Sad—Neutral" refers to inducing a sad mood, and evaluating its reduction in a neutral ambience.

Group	Age	Session 1	Session 2
1 (n = 9; 5 female)	M = 76.8, SD = 8.8	Sad—Neutral	Anxious—Cozy
2 (n = 9; 4 female)	M = 78.5, SD = 6.9	Sad—Activating	Anxious—Neutral
3 (n = 10; 5 female)	M = 80.5, SD = 8.2	Anxious—Cozy	Sad—Neutral
4 (n = 10; 5 female)	M = 79.2, SD = 7.7	Anxious—Neutral	Sad—Activating

The participants entered the experimental room with a neutral lighting. After signing the informed consent the sensors for the physiological measurements were applied. Hereafter a fifteen minutes waiting period started to ensure that the preparations of the physiological measurements did not influence the baseline mood measurements. During the first five minutes of this waiting period, the experimenter explained the setup and the questionnaire scales to the participants. After the fifteen minutes waiting period, the participants verbally indicated their subjective mood on that moment based on the SAM scales. The lighting was then dimmed to a low level (i.e., 40 lx) and the mood inducing short movie was displayed on the television screen. After the mood induction, the second SAM measure was collected. Hereafter the lighting was changed to a neutral, cozy or activating setting, depending in which group and session the participant was in. From that moment, the participants indicated their actual mood every two minutes with the SAM scale. After the seventh mood measurement (i.e., after 10 minutes), a short break was incorporated. The second session followed a procedure similar to the first session with only a shorter relaxing period for the baseline measurement (i.e., 5 minutes). After the experiment, participants were thanked for their participation and debriefed. They received a coupon of 20 euros for their participation. The whole experimental procedure is schematically shown in Table 2.

**Table 2:** Schematic overview of the experimental procedure.

	Session 1	Session 2
*Neutral lighting*	Preparations physiological measurements	Break
	*waiting period (15 min)*	*waiting period (5 min)*
	Baseline mood (SAM)	Baseline mood (SAM)
*Film lighting*	Mood induction (Sad film)	Mood induction (Anxious film)
	Mood after MI (SAM)	Mood after MI (SAM)
*Cosy/activating or neutral lighting*	*waiting period (2 min)*	*waiting period (2 min)*
	2 min (SAM)	2 min (SAM)
	*waiting period (2 min)*	*waiting period (2 min)*
	4 min (SAM)	4 min (SAM)
	*waiting period (2 min)*	*waiting period (2 min)*
	6 min (SAM)	6 min (SAM)
	*waiting period (2 min)*	*waiting period (2 min)*
	8 min (SAM)	8 min (SAM)
	*waiting period (2 min)*	*waiting period (2 min)*
	10 min (SAM)	10 min (SAM)

## Results

Equipment and recording errors led to missing data for the physiological measurements. The sample sizes for each of the dependent measures are depicted in Table 3. We found no carrying-over effects between the first and second session; the baseline scores from the first session were not significantly different from the baseline scores of the second session for all the measures, as tested with paired-samples t-tests.

**Table 3:** Sample sizes for the different dependent measures for each ambience group.

Ambience group	Pleasure and arousal ratings	Skin conductance response	Heart rate and Heart rate variability
Sad movie-> neutral ambience	n = 19	n = 18	n = 17
Sad movie-> activating ambience	n = 19	n = 18	n = 18
Anxious movie-> neutral ambience	n = 19	n = 18	n = 17
Anxious movie-> cozy ambience	n = 19	n = 19	n = 16

### Effectiveness of mood induction

To investigate the influence of the sad and anxious mood induction on the affective ratings and physiological measures, we tested a series of general linear models with gender and ambience group as between-subjects factors and mood induction (pre/post) as the within-subjects factor. For the physiological measures we compared the median values of the last 120 seconds of the baseline with the median values of last 120 seconds of the mood induction.

**Fig 4:**
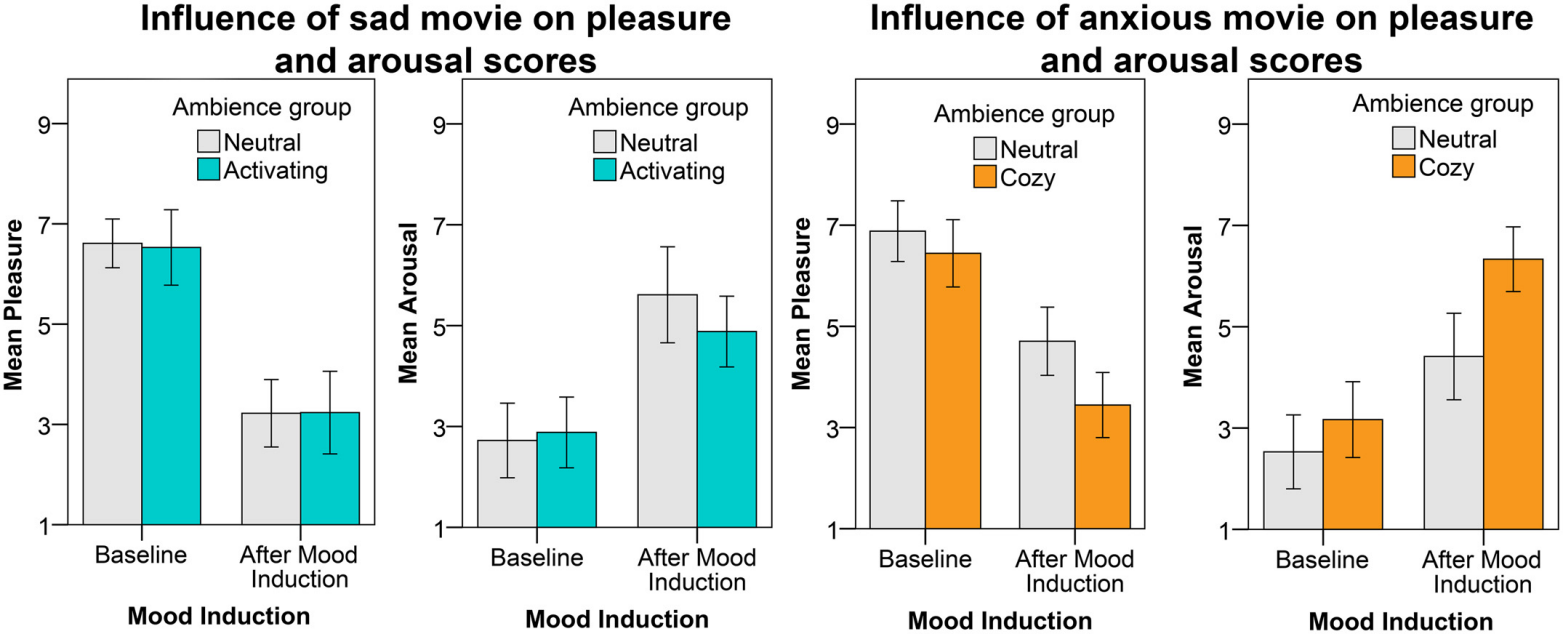
Average pleasure and arousal scores before and after the sad movie (left) and anxious movie (right). The different bars represent the different ambience groups. The error bars reflect the 95% CI.

Fig 4 presents the subjective pleasure (left) and arousal (right) scores before and after the mood induction for both the sad and anxious movie. For the sad movie we found that the pleasure ratings were significantly reduced after the mood induction (F(1, 34) = 120.92, p < .001, η^2^ = .78) as intended. Also the effect of gender reached significance for pleasure (F(1, 34) = 6.33, p = .017, η^2^ = .16); further analyses revealed that males scored higher on pleasure at baseline (M = 7.11) and after the sad movie (M = 4.21) than females (at baseline M = 6.26, and after the sad movie M = 2.95). In contrast with our expectations the SAM arousal ratings were significantly increased by the mood induction (F(1, 34) = 37.27, p < .001, η^2^ = .52). We found also here a gender effect (F(1, 34) = 8.27, p = .007, η^2^ = .20). Further analysis revealed that females scored higher on arousal at baseline (M = 3.21) and after the sad movie (M = 5.53) than males (at baseline M = 2.11, and after the sad movie M = 4.32). For the physiological measures a significant effect of the sad mood induction on SCR and HR was found. In line with the SAM arousal ratings, also the SCR significantly increased during the mood induction (F(1, 32) = 4.44, p = .043, η^2^ = .12) from M = 4.09 at baseline to M = 4.34 during mood induction. Also the HR increased significantly (F(1, 31) = 4.89, p = .034, η^2^ = .14) from M = 66.58 at baseline to M = 67.40 during mood induction. Furthermore a gender x ambience group interaction effect was found on HR (F(1, 31) = 9.44, p = .004, η^2^ = .23); further analysis revealed that the females in the neutral ambience group had a higher HR (M = 74.1) than the male participants (M = 60.6), whereas a reversed effect was found for the activating ambience group (females M = 63.2, and males M = 70.9).

After the anxious mood induction the (self-reported) pleasure ratings were significantly reduced as expected (F(1, 34) = 76.38, p < .001, η^2^ = .69). A significant effect of ambience group on pleasure was found (F(1, 34) = 6.17, p = .018, η^2^ = .15). As can be seen in Fig 4, the elderly in the cozy ambience group scored lower on pleasure at baseline and after the mood induction. Also the effect of gender reached significance (F(1, 34) = 9.53, p = .004, η^2^ = .22); further analysis revealed that the males scored again higher on pleasure before (M = 7.32) and after the anxious movie (M = 5.05) than the females (at baseline M = 6.26, and after the anxious movie M = 3.74). The arousal ratings were increased after the anxious mood induction (F(1, 34) = 76.38, p < .001, η^2^ = .65). The mood induction and ambience group interaction was also significant (F(1, 34) = 4.97, p = .032, η^2^ = .13). As can be seen in Fig 4 (right figure), the arousal scores of the elderly in the cozy ambience group increase more after the anxious movie than the arousal scores of the elderly in the neutral ambience group. The effect of ambience group on arousal was also significant (F(1, 34) = 8.45, p = .006, η^2^ = .20). The elderly in the cozy ambience group scored higher on arousal at baseline and after the mood induction (as can be seen from Fig 4). For the physiological measures, significant effects of the anxious mood induction were found on SCR and HR. The SCR significantly increased from baseline (M = 4.45) to after the mood induction (M = 4.78) in line with our expectations and the SAM ratings (F(1, 33) = 5.81, p = .022, η^2^ = .15). For HR we found a significant gender x ambience group interaction effect (F(1, 29) = 10.35, p = .003, η^2^ = .26); further analysis revealed that males in the neutral ambience group had a higher HR (M = 70.8) than the females (M = 62.3), whereas a reversed effect was found for the cozy ambience group (males M = 62.0, and females M = 75.6).

### Influence of lighting on mood

To analyze the influence that the activating and cozy ambiences had on mood (with respect to that of the neutral ambience), we monitored the evolution of the affective measures, starting from the moment the lighting was changed from the movie lighting setting to the cozy, activating or neutral setting (see Table 2). In particular, we resorted to what we call “Affective change scores”. Affective change scores were calculated for every moment after the mood induction and for every participant by subtracting the affective rating measured at the end of the mood induction procedure from the affective rating measured after each of the five waiting periods (i.e., ‘2 min’, ‘4 min’, ‘6 min’, ‘8 min’, and ‘10 min’). For the physiological measures, affective change values were calculated by subtracting the median value of the signal over the last 120 seconds of the mood induction procedure from the median value measured during the 120 seconds of each of the five waiting periods (i.e., ‘2 min’, ‘4 min’, ‘6 min’, ‘8 min’, and ‘10 min’).

A general linear model was tested for each dependent variable (i.e., the affective change scores for SAM and the affective change values for the physiological measures). Gender and ambience group were included as between-subjects factors and ‘time’ (as discretized in the five waiting periods described above) was included as a within-subjects factor. Since the previous analysis (on the effectiveness of mood induction) revealed that the ambience groups were sometimes influenced in a different way by the mood induction, we also calculated for each participant and for each dependent variable the reaction to the movie. The latter was then included as covariate in the analyses. The reaction to the movie was calculated by subtracting the rating at baseline from the rating after mood induction for the affective scores, and by subtracting the median values of the last 120 seconds of the baseline from the median value of the last 120 seconds of the mood induction for the physiological measures. The covariate was significant in all analyses described below.

### Influence of activating ambience on sad mood

**Fig 5:**
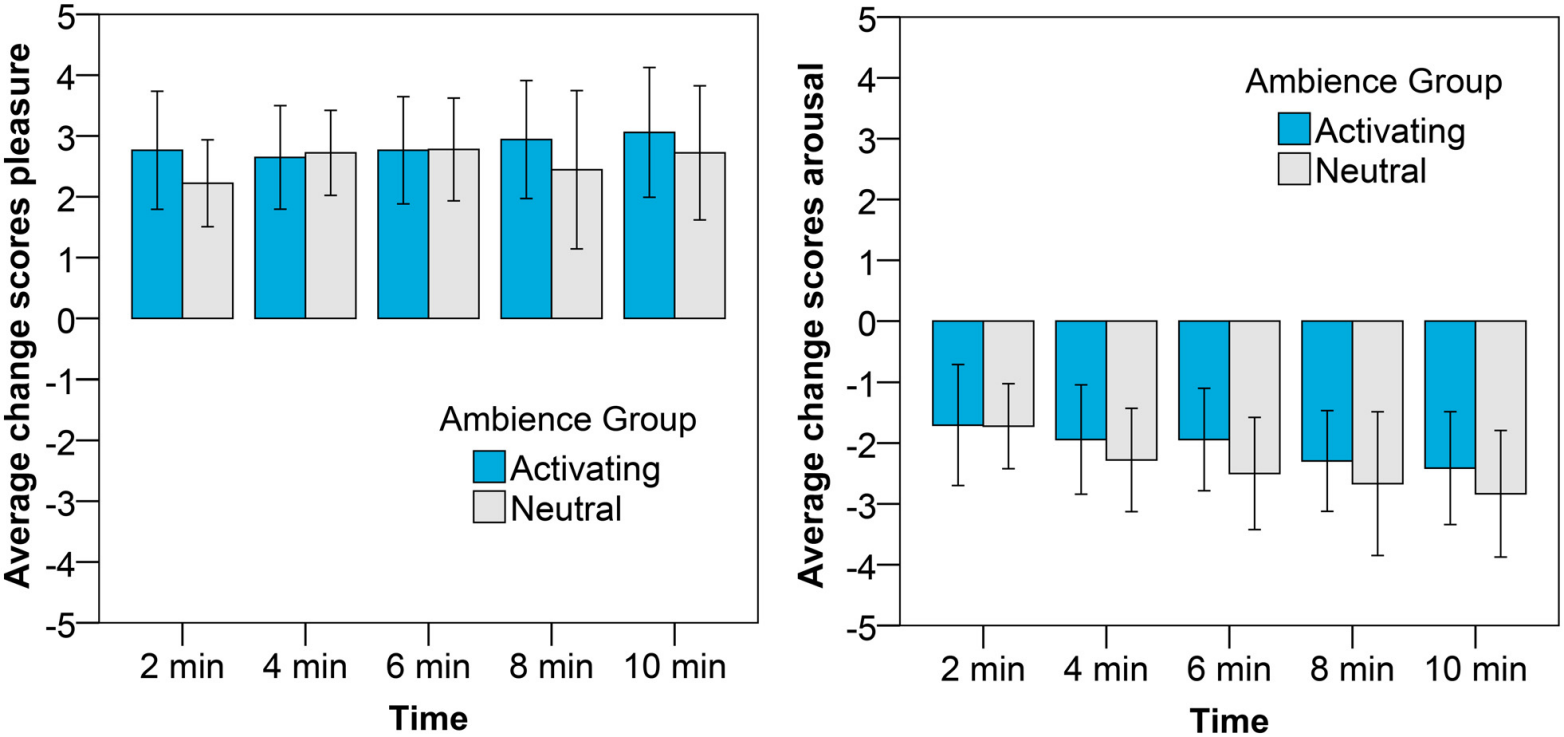
Average change scores with the error bars reflecting the 95% CI for pleasure (left) and arousal (right) after the sad mood induction. The different bars represent the ambience to which the participants were exposed.

Fig 5 presents the subjective pleasure and arousal change scores for participants exposed to the neutral and activating ambiences after the sad mood induction. The pleasure change scores are slightly higher for the participants in the activating ambience than for the participants in the neutral ambience; this effect is, however, not significant. The arousal change scores are reduced more under the neutral lighting than under the activating lighting; however also this effect is not significant. No significant effect of gender or time on the affective change scores, nor a significant interaction were found.

**Fig 6:**
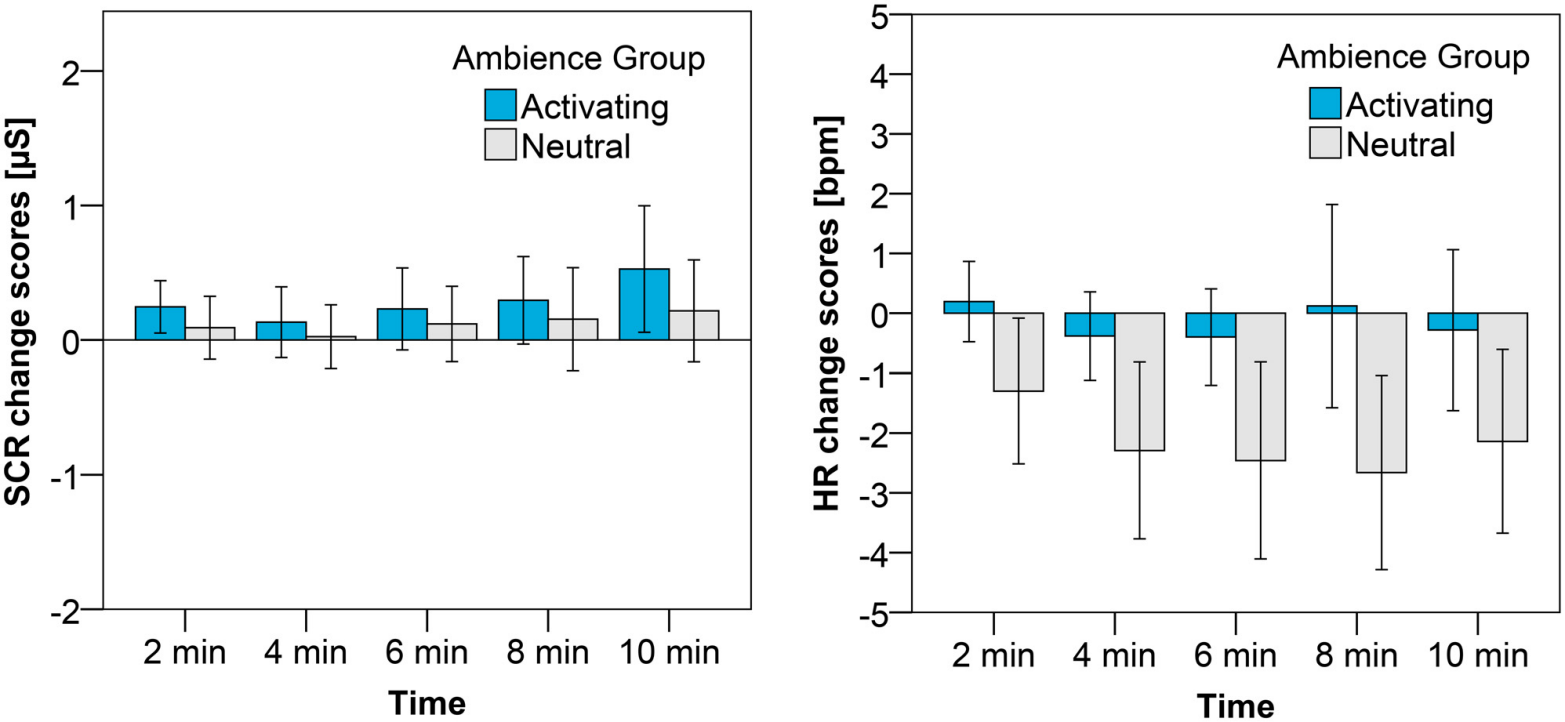
Average change scores with the error bars reflecting the 95% CI for SCR (left) and HR (right) after the sad mood induction. The different bars represent the ambience to which participants were exposed.

With respect to the physiological measures (shown in Fig 6) we found significant differences between the activating and neutral ambience for both the SCR and HR measures. In line with our expectations, the SCR of the participants immersed in the activating ambience increased more compared to the SCR of the participants immersed in the neutral ambience (F(1, 31) = 4.30, p = .046, η^2^ = .12). Also the effect of time reached significance (F(2.4, 75.7) = 6.21 p = .002, η^2^ = .17); the SCR increased during the 10 minutes after mood induction for both the participants in the activating ambience and in the neutral ambience (see also Fig 6). The effect of ambience on HR was also significant (F(1, 30) = 5.72, p = .023, η^2^ = .16). The heart rate decelerated for the participants in the neutral ambience, however not for the participants in the activating ambience (see again Fig 6), as such indicating a higher physiological arousal in the activating ambience. No significant effects were found on the heart rate variability measurements.

### Influence of cozy ambience on anxious mood

**Fig 7:**
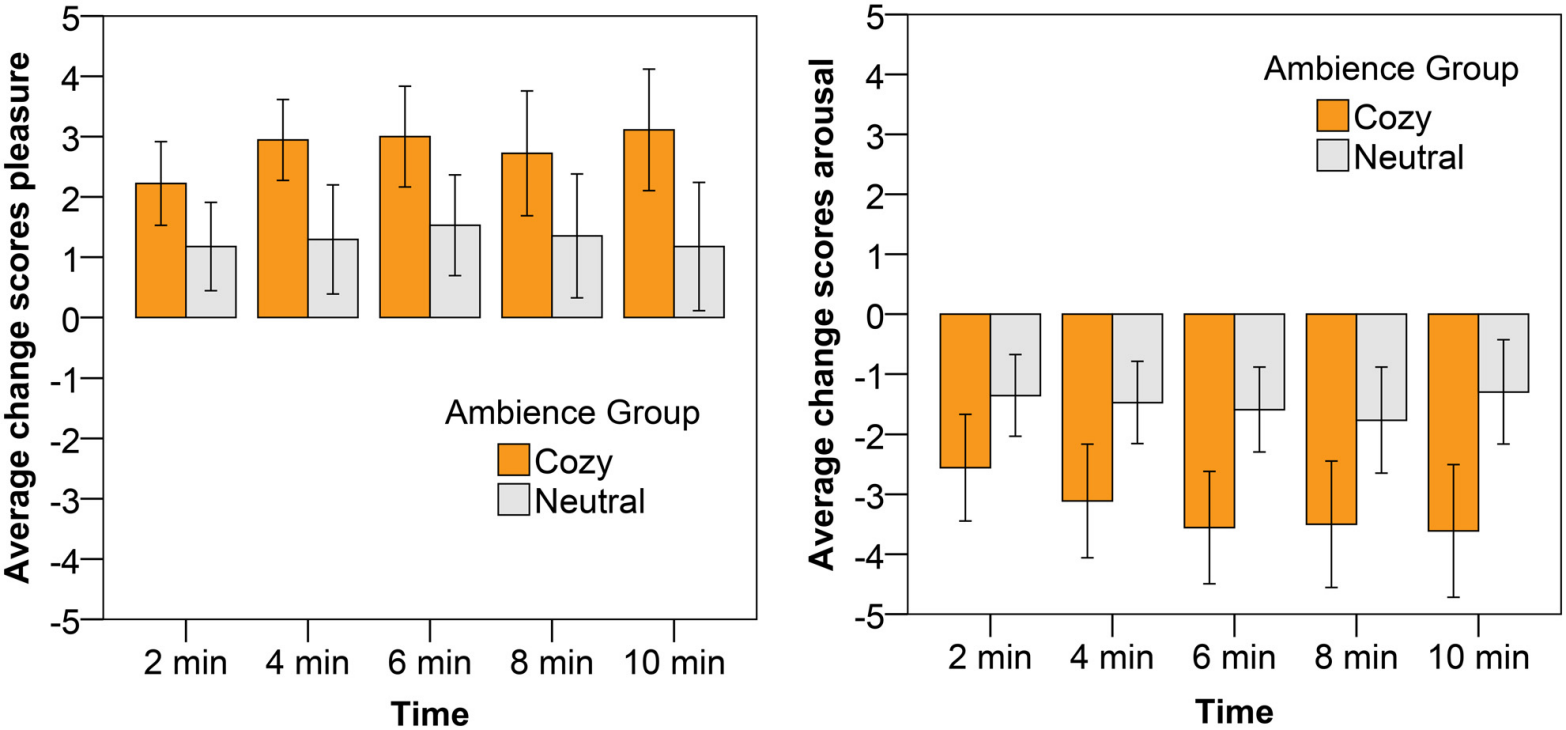
Average change scores with the error bars reflecting the 95% CI for pleasure (left) and arousal (right) after the anxious mood induction. The different bars represent the different ambiences in which participants were immersed.

**Fig 8:**
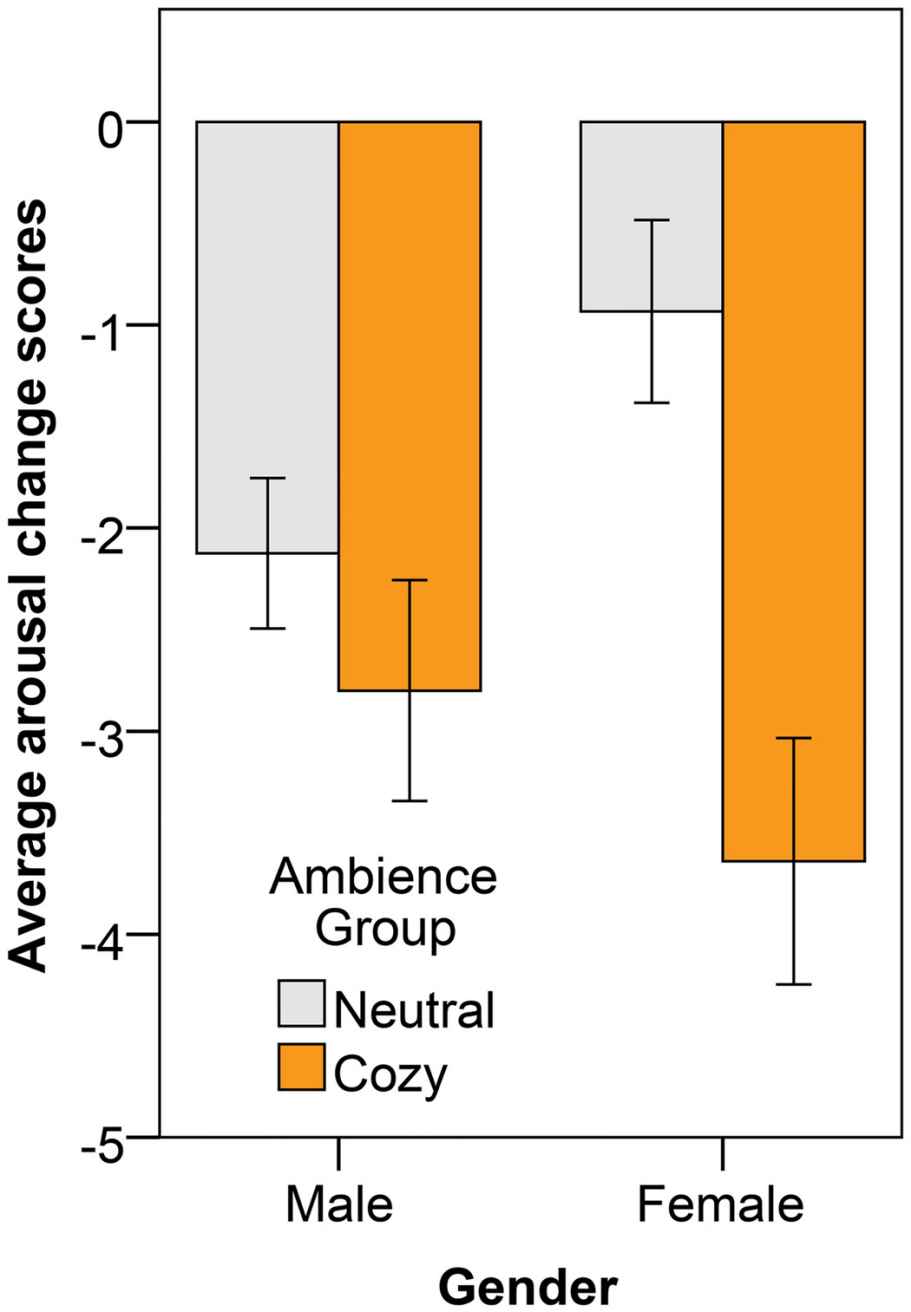
Average arousal change scores (also averaged over time), showing also the 95% CI as error bars, after the anxious mood induction for the male and female participants separately. The different bars represent the different ambiences in which participants were immersed.

The subjective pleasure and arousal change scores for all measurements after the anxious mood induction are presented in Fig 7, for both the cozy and the neutral ambience group. The pleasure change scores are higher for participants exposed to the cozy lighting as compared to participants immersed in a neutral lighting; indeed, we found a significant effect of ambience on pleasure (F(1, 33) = 5.68, p = .023, η^2^ = .15). The arousal scores were significantly more reduced under cozy lighting than under neutral lighting (F(1, 33) = 6.10, p = .019, η^2^ = .16). The effect of ambience group x gender on arousal was also significant (F(1, 33) = 4.37, p = .044, η^2^ = .12); the difference in arousal change score between the neutral and cozy ambience was larger for the female participants than for the male participants (as is illustrated in Fig 8).

**Fig 9:**
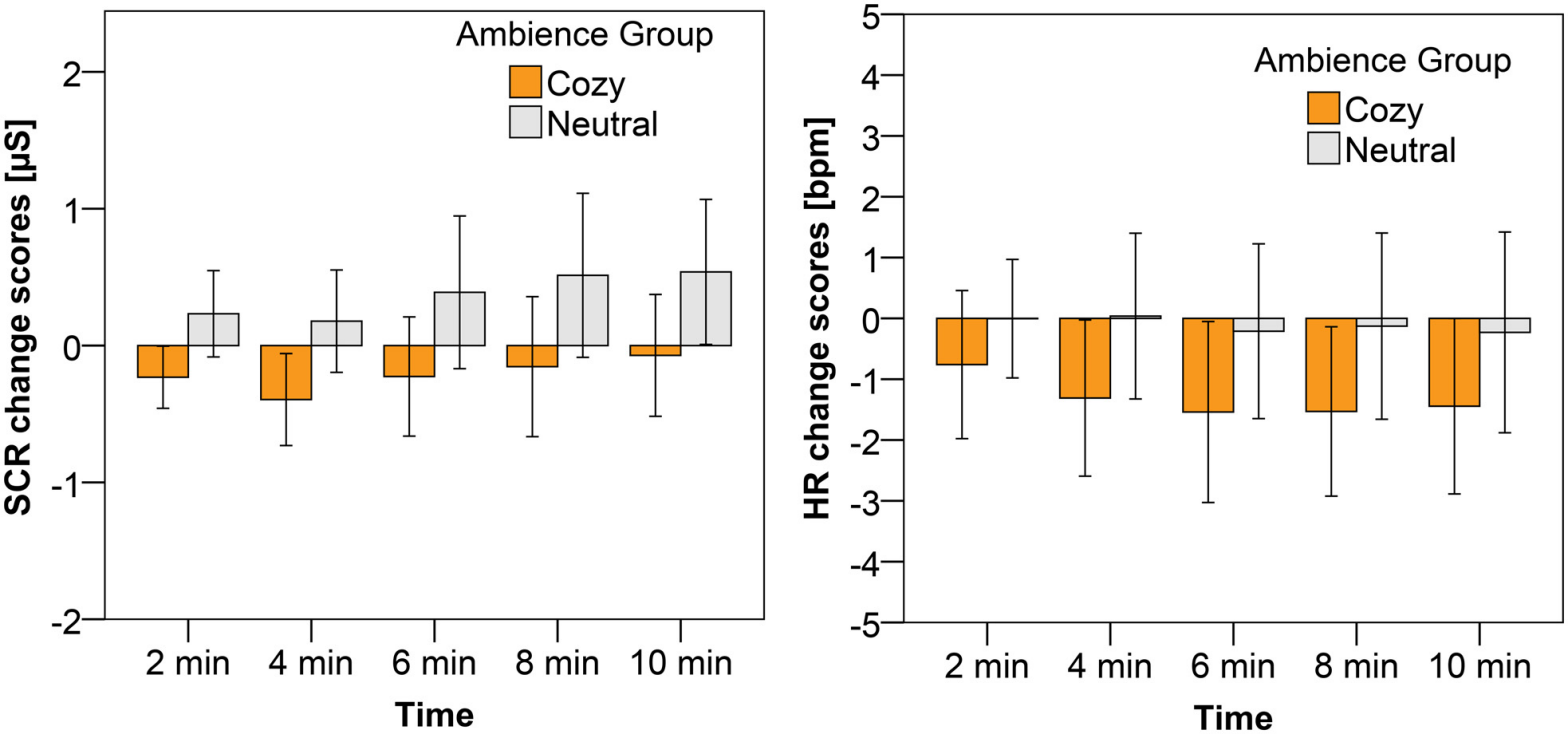
Average change scores, including the 95% CI as error bars, for SCR (a) and HR (b) after the anxious mood induction. The different bars represent the different ambiences in which participants were immersed.

With respect to the physiological measures, we found a significant effect of ambience on SCR (F(1, 32) = 4.43, p = .043 η^2^ = .12). As expected and in line with the SAM arousal ratings, the SCR affective change values of the participants in the cozy ambience group were significantly lower than the SCR affective change values of the participants in the neutral ambience group (as is illustrated in Fig 9). The HR also decelerated after the anxious mood induction for the participants in the cozy ambience, while that was not the case for the participants in the neutral ambience group (see also Fig 9). This effect was however not significant. No significant effects were found on the HRV measures.

## Discussion

In this study, we investigated whether atmospheres created with lighting (that is, ambiences with a clearly recognized affective connotation) had the potential to improve a negative mood of elderly. Specifically, we studied whether these affective ambiences were more effective in countering negative moods than neutral ambiences, in terms of both increasing the elderly's mood valence and stabilizing the arousal towards a more neutral level. According to both self-reported and physiological measures, the low arousing, pleasant ambience (i.e., *cozy*) was found to be more effective in reducing a high arousing negative mood state (i.e., anxiety) than the neutral ambience. The high arousing, pleasant ambience (i.e., *activating*) was found to increase the physiological arousal of sad elderly more than the neutral ambience, but this finding was not supported by changes in SAM arousal ratings.

The effectiveness of the ambiences in countering negative moods was tested under controlled conditions in a dedicated laboratory environment. The test participants (healthy elderly people) were on average in a pleasant (M = 6.8 on a scale from 1 to 9) low arousing (M = 3.0 on a scale from 1 to 9) mood state. We induced two negative mood states with a sad and anxious movie excerpt. Both the anxious and the sad movie were successful in inducing negative valence; however, both movies also induced arousal. The anxious movie induced a high arousing negative mood state as intended and in line with previous research [52], as verified through the analysis of SAM arousal ratings and as supported by an increased electrodermal response. The sad mood induction was intended to induce a low arousing negative mood state. In contrast with previous research [52], we found that the movie excerpt from ‘The champ’ also induced arousal. A possible explanation for this is that previous results were derived from observing mood induction on younger adults. Our reference group instead consisted of elderly, for which the response to mood induction may be different. Literature suggests that elderly may experience more arousal when the sad mood induction is very relevant to their age group [62,63]. When watching sad movies related to family loss, older people’s physiological arousal indeed increased more than that of younger people [62,63]. In addition, also other affective responses may be induced on top of sadness; Kunzmann and Gruhn [62] found that a family loss movie also induced higher arousing mood states, as anxiety and anger, especially in older participants.

Despite the random assignment of the participants, while counterbalancing gender, we found group differences in the reaction to the movies. Especially the elderly assigned to the cozy ambience group had a more pronounced reaction to the anxious movie compared to the elderly assigned to the neutral ambience group (note that both groups viewed the movie in the same lighting condition). The more pronounced reaction might explain the differences we found after the mood induction between the cozy and neutral ambience. To control for this effect, we calculated the reaction to the mood induction for each participant separately (and also for each dependent measure separately), and included it as covariate in the analysis. Part of the differences found between the cozy and neutral ambiences were indeed explained by the covariate. However we still found that the elderly in the cozy ambience reported significantly higher pleasure ratings and significantly lower arousal ratings than the elderly in the neutral ambience. The SCR of the participants was also significantly reduced in the cozy ambience compared to the neutral ambience, even when the covariate was included in the analysis. The effect of HR however was almost completely explained by the covariate, underpinning the importance of including the covariate in the analyses.

We found a main effect of gender on the self-reported pleasure and arousal ratings after mood induction and an interaction between ambience and gender on HR. These effects could be completely explained by baseline differences, and so, the female and male participants did not react differently to the affective movies (i.e., no significant mood induction x gender interactions were found). Some studies suggest that female participants are more susceptible to mood influences than male participants [64,65]. These studies are however performed with young adults; the few studies which investigated the effect of mood induction on elderly participants did not find gender effects [66–68]. We however also found a significant effect of gender on the reaction to the cozy versus the neutral ambience. Although the cozy ambience reduced the arousal scores of both the female and male participants as compared to the neutral ambience, this reduction was far more effective for the female participants. Knez and colleagues even found that lighting can have reversed effects on the mood of females compared to males [39,40].

The activating ambience was found to be physiologically arousing compared to the neutral ambience. No significant effects were found, though, on the self-reported levels of arousal between the neutral and activating ambience; both participants immersed in the activating ambience and participants exposed to the neutral ambience reported a similar reduction in arousal during the waiting periods after the mood induction. The lack of effectiveness of the activating ambience on the self-reported arousal may be due to a number of reasons. First, the setup of the experiment might have worked against the psychological effect of the activating ambience. In our protocol, participants went through five waiting periods of two minutes each, during which they did nothing while sitting on a couch. On top of this, they were asked to limit their movements not to hamper the recording of physiological measurements. Thus, it may be the case that participants experienced the activating effect of the ambience, however, the experimental task prevailed a more active stance and thus a more active feeling. Second, it may be the case that the period of ten minutes was too short to realize a positive activating mood state. Knez and colleagues [39–41] argue that longer exposure times of several hours, are needed to generate consistent effects of indoor lighting on mood. Although the results of the cozy ambience suggest that consistent effects can be reached in short periods, it might require longer exposure times to positively energize people.

We focused on the psychological effects of lighting on mood, however our ambiences did not violate the known biological effects of lighting. The low arousing cozy ambience had lighting with a low illuminance and with a higher spectral density at the higher wavelengths (resulting from a lower CT of the functional white lighting and orange colored accent lighting). The high arousing activating ambience had a higher illuminance with a higher spectral density at shorter wavelengths (resulting from with a higher CT of the functional white lighting and cyan colored accent lighting). So, if the lighting would have had any biological effect on mood, it would have been in accordance to the psychological effect. The illuminance levels, however, even in the activating ambience, are generally too low to reach a direct biological arousing effect during day time.

We did not find any significant effect of atmosphere on the HRV measures. A possible explanation may be found in the timeframes of 120 seconds for the analysis of the HRV data, which may be too short. Periods of 5 minutes are recommended for short-term HRV recordings [60]. Such a longer recording time allows a more accurate calculation of the low frequency component of HRV in the frequency domain (HRV-LF), which allows the calculation of normative values of HRV-HF and the HRV-HF/HRV-LF ratio. The setup of the experiment, however, did not allow the selection of longer timeframes for the analysis of the physiological data because we wanted to measure the evolution of the mood over short time periods, to avoid longer waiting times possibly inducing boredom or frustration in the participants.

This investigation also has limitations, which should be acknowledged. First, we tested the effect of the ambiences on induced negative moods and not on genuine negative moods. Although research revealed that induced moods have similar properties and similar effects on cognition and behavior as real negative moods [68], we know that the induced moods diminish quickly after the mood induction. The effectiveness of the ambiences should be further tested with elderly which are actually experiencing negative moods. Secondly, we only tested short-term effects of the ambiences, while long-term effects are important to be investigated in order to be able to claim that these ambiences are fully beneficial for elderly. The effects of longer and repeated exposure to ambiences still need to be determined. Thirdly, we used the Self-Assessment Manikin (SAM) for measuring the self-perceived mood state of the elderly. This method is quick and relatively low on cognitive effort, however at the cost of more detailed mood information. As a result, we have no information on whether the elderly for instance experienced mixed affective states after the mood induction, as might be the case after the sad mood induction. It is also unknown at this stage whether the setup of the experiment (with the five waiting periods) induced negative affective states like boredom or frustration.

Another limitation of the current study is that we considered two ambiences which were on average found to be cozy or activating by our target group. However, within our group of participants large variations in intensity sensitivity and color perception may exist, due to the age-related deterioration of the visual system [69]. Individual preferences and gender differences may also have an impact on the optimality of ‘cozy’ and ‘activating’ ambiences. The usage of an ambience considered ‘cosy’ or ‘activating’ by an average elderly may therefore result in suboptimal effectiveness of the ambience.

Finally, we did not control for the contribution of separate lighting characteristics to the measured mood effect, since the ambiences differed simultaneously in illumination, color temperature and the colors used for the accent lighting. In this study, though, we were interested in investigating if ambiences with a clear affective meaning could change the mood of people in the direction of the affective meaning of the ambience. Next steps will be dedicated to link lighting characteristics to their effect on mood, starting from the knowledge existing on the relationship between lighting characteristics and atmosphere perception.

## Conclusion

In this study, we showed that we can improve the mood of elderly by exposing them to lighting ambiences with a clear, positive affective meaning. Sad elderly which were immersed in a positive high arousing (i.e., activating) ambience were physiologically more aroused than sad elderly which were immersed in a neutral ambience. The anxious elderly could be effectively calmed with a cozy ambience. Indeed, anxious elderly immersed in a cozy ambience were physiologically less aroused and reported lower arousal scores and higher pleasure scores as compared to anxious elderly immersed in a neutral ambience. First assessing how elderly perceive the atmosphere of the ambiences [12] was found to be an efficient and reliable method to find the lighting settings, which could effectively induce the desired mood change. To fully validate our approach, the effectiveness of the ambience in improving mood should be further tested with longer exposure times and with elderly with genuine negative moods. Once their effectiveness is proven, we expect these ambiences to be beneficial also in contexts different from the care center one, such as in hospital rooms.
